# The inhibiting effect of neural stem cells on proliferation and invasion of glioma cells

**DOI:** 10.18632/oncotarget.20270

**Published:** 2017-08-14

**Authors:** Jing An, Hanqi Yan, Xingxing Li, Ruolan Tan, Xinlin Chen, Zhichao Zhang, Yingfei Liu, Pengbo Zhang, Haixia Lu, Yong Liu

**Affiliations:** ^1^ Institute of Neurobiology, School of Basic Medical Sciences, Xi’an Jiaotong University Health Science Center, Xi’an, Shaanxi 710061, P.R.China; ^2^ Department of Human Anatomy and Histoembriology, School of Basic Medical Sciences, Xi’an Jiaotong University Health Science Center, Xi’an, Shaanxi 710061, P.R.China; ^3^ Department of Anesthesia of The Second Affiliated Hospital, Xi’an Jiaotong University Health Science Center, Xi’an, Shaanxi 710004, P.R.China

**Keywords:** neural stem cells, glioma, inhibiting effect, proliferation, invasion

## Abstract

The invasive and infiltrative nature of tumor cells leads to the poor prognosis of glioma. Currently, novel therapeutic means to eliminate the tumor cells without damaging the normal brain tissue are still strongly demanded. Significant attentions had been paid to stem cell-based therapy and neural stem cell (NSC) had been considered as one of the efficient delivery vehicles for targeting therapeutic genes. However, whether the NSCs could directly affect glioma cells remains to be seen. In this study, both rat and human glioma cells (C6 and U251) were co-cultured with normal rat embryonic NSCs directly or in-directly. We found the survival, proliferation, invasion and migration of glioma cells were significantly inhibited, while the differentiation was not affected in the *in vitro* co-culture system. In nude mice, although no significant difference was observed in the tumor growth, survival status and time of tumor-bearing mice were significantly promoted when U251 cells were subcutaneously injected with NSCs. In coincidence with the suppression of glioma cell growth *in vitro*, expression of mutant p53 and phosphorylation of AKT, ERK1/2 decreased while the level of caspase-3 increased significantly. Our results suggested that normal NSCs could possess direct anti-glioma properties via inhibiting the glioma cell viability, proliferation, invasion and migration. It could be a very promising candidate for glioma treatment.

## INTRODUCTION

Glioma is the most common and malignant brain tumor in the central nervous system (CNS). The invasive and infiltrative nature of tumor cells, as well as the difficulties of effective treatment leads to the very poor prognosis. Although several therapeutic strategies, including surgery, radiotherapy, chemotherapy and even the gene therapy have been widely used, the 5-year survival rate of glioma is still less than 10% [1–3]. Therefore, novel therapeutic means to eliminate invasive tumor cells without damaging the normal brain tissue are still urgently required.

Significant attention has been paid to stem cell-based therapy for glioma treatment [4–12]. Not only the tumor stem cell (TSC) has been considered as the specific target for tumor therapy [4–7], normal stem cell also has been used as an efficient vehicle to deliver anti-tumor substances [8–12]. Neural stem cell (NSC) is a group of immature cells in the nervous system [13, 14]. It holds much promise for the treatment of spinal cord injury, stroke, and many neurodegenerative diseases [15–17]. Moreover, it also has several advantages, including the ability to cross the blood brain barrier, no toxicity to normal tissue, and the tumor-tropic property, that ensure its use in delivering anti-tumor agents [11, 12, 18–26]. So far, many molecules, like bone morphogenetic protein (BMP), herpes simplex virus thymidine kinase/ganciclovir (HSVtk/GCV) system, cytokines and cytosine deaminase, ect, have been successfully delivered by NSCs and reduced the progress of glioma. However, the capacity of NSC to deliver anti-tumor substances is only one aspect of NSC-based therapy in treatment of tumor. Whether NSCs could directly affect glioma cells still remains to be seen.

Interestingly, Glass R et al., have showed that normal neural precursors possessed age dependent anti-tumorgenic potency [27]. It indicated that NSCs did perform some direct effects on glioma cells. In order to further investigate the direct effect of NSC on biological behaviors of glioma cells, in current study, both rat glioma cell C6 and human glioma cell U251 were co-cultured with normal rat embryonic NSCs, respectively. Then the biological behaviors, including the survival, proliferation, differentiation, invasion and migration of glioma cells were observed in the *in vitro* co-culture system. Then the tumor growth and survival status of tumor-bearing nude mice were investigated after the subcutaneous injection of U251 with NSCs. In addition, the phosphorylation of MAPK, PI3K/AKT and the expression of mutant p53, caspase-3 were detected to investigate the possible mechanisms.

## RESULTS

### Survival of tumor cells in NSCs growth medium

**Figure 1:**
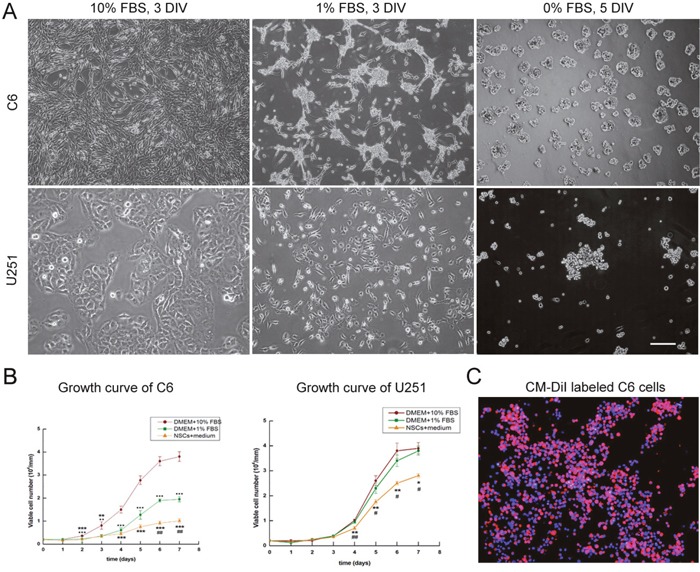
Culturing tumor cells in NCS growth medium **(A)** C6 and U251 cells were cultured in DMEM with different concentrations of FBS. **(B)** Diverse growth of C6 and U251 cells in different mediums. **(C)** CM-DiI labeled C6 cells. DAPI used for cell nuclei staining showed in blue, CM-DiI was in red. Scale bars = 100 μm (A) and = 50 μm (C), DIV: days *in vitro*. *p <0.05.

In order to set up the co-culture system, C6 and U251 cells were adaptively cultured in DMEM medium with 1% fetal bovine serum (FBS) and then serum free NSCs growth medium. Both of C6 and U251 cells grew adherently in the medium with 1% FBS and shared similar morphology with that in the tumor cell medium which contained 10% FBS. However, in the medium without serum, C6 and U251 cells grew into 3-dimensional spheres (Figure 1A). Diverse growth of tumor cells in different mediums was observed (Figure 1B, *p*<0.05). Before co-cultured with NSCs, tumor cells were labeled with CM-DiI (Figure 1C) and the efficiency of labeling, detected under fluorescent microscope, was approximately 95%.

### Survival and proliferation of tumor cells after co-cultured with NSCs

**Figure 2:**
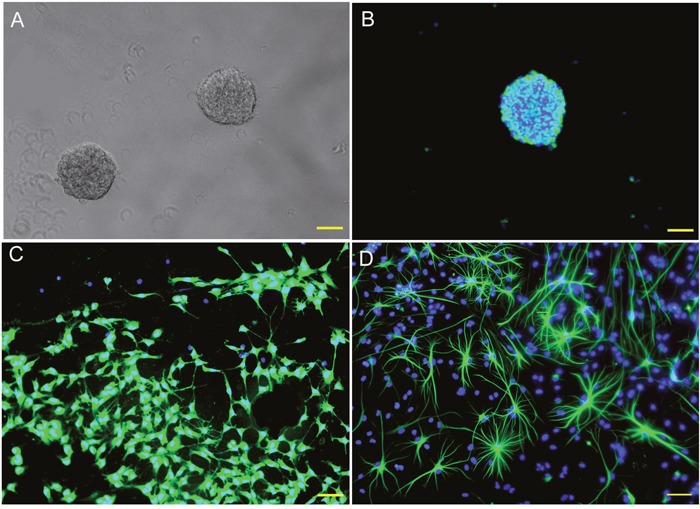
Culturing and identification of rat NSCs **(A)** Neurospheres in different sizes observed in the culture system at 7 DIV. **(B)** Most of the cells in the neurosphere are nestin positive NSCs. **(C)** Some of those cells differentiated into β tubulin III positive neurons in differentiation medium. **(D)** Some of the cells differentiated into GFAP positive astrocytes in differentiation medium. DIV: days *in vitro*; DAPI were used for cell nuclei staining and showed in blue. Nestin (B), β tubulin III (C) and GFAP (D) were used for NSCs, neurons and astrocytes identification and showed in green. Scale bar = 50 μm.

**Figure 3:**
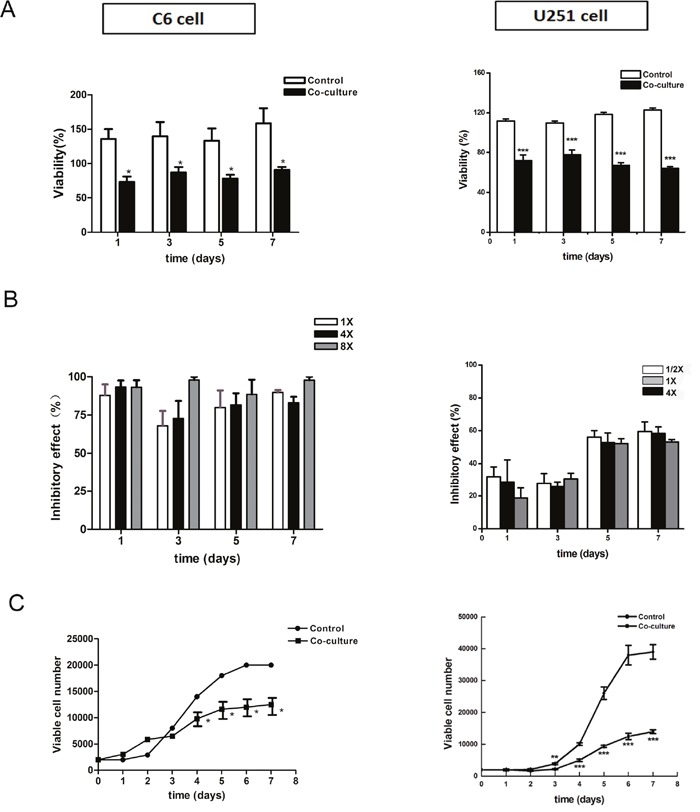
Survival and growth of tumor cells in the directly co-culture system **(A)** CCK-8 assay showed that cell viability significantly reduced after co-culturing. **(B)** Inhibitory rate calculation showed that there was no significant difference between different groups, which contained different numbers of NSCs. **(C)** Growth curve of CM-DiI labeled C6 and U251 cells showed the significantly reduction of tumor growth. 1×, 4× and 8× mean the ratio of C6 cells verse NSCs were 1 to 1, 1 to 4 and 1 to 8, respectively. 1/2×, 1× and 4× mean the ratio of U251 cells verse NSCs were 2 to 1, 1 to 1 and 1 to 4, respectively.*p<0.05, **p<0.01,***p<0.001.

Rat embryonic NSCs were cultured and identified as previous (Figure 2). CM-DiI labeled C6 and U251 cells were co-cultured with different numbers of NSCs in NSC growth medium. Tumor cell viability, detected by CCK-8 assay, significantly decreased (Figure 3A, *p*<0.05). Regarding the inhibitory rate, there was no significant difference between different groups which contained different cell proportions (Figure 3B, *p*>0.05). Furthermore, the growth curves of tumor cells were plotted based on the number of CM-DiI labeled C6 and U251 cells at different time points. The result showed the growth of C6 and U251 cells were significantly inhibited (Figure 3C, *p*<0.05).

### Differentiation of tumor cells after co-cultured with NSCs

**Figure 4:**
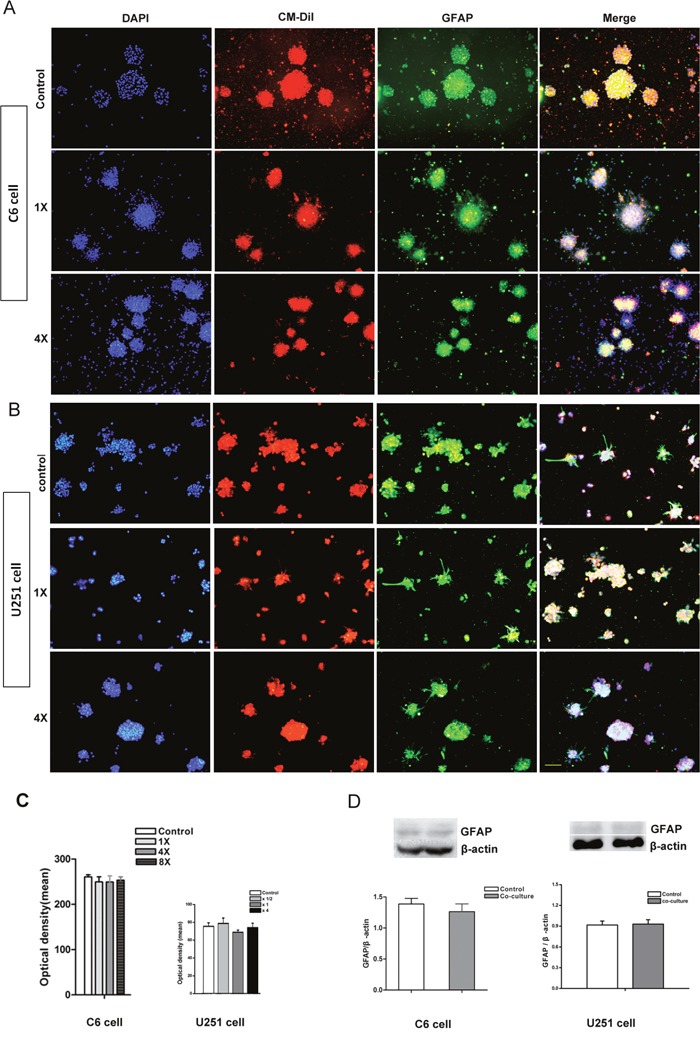
Differentiation of tumor cells in the directly co-culture system **(A)** Immunocytochemistry staining showed that almost all of the CM-DiI labeled C6 cells were GFAP positive. **(B)** Immunocytochemistry staining showed that almost all of the CM-DiI labeled U251 cells were GFAP positive. **(C)** The intensity of GFAP fluorescent measured by image-pro plus 5.0 software showed that there was no significant difference between different groups. **(D)** Western blot quantifying the GFAP protein in whole co-culture system showed that there was no significant difference between different groups. DAPI used for cell nuclei staining showed in blue, CM-DiI showed in red and GFAP showed in green. 1× and 4× mean the ratio of tumor cells verse NSCs were 1 to 1 and 1 to 4. Scale bar = 50 μm.

After direct co-culture for 7 days, cell spheres were fixed and GFAP immunocytochemistry staining was performed. The result showed that almost all of the CM-DiI labeled C6 and U251 cells were GFAP positive. There was no significant difference between different groups (Figure 4A–4C, *p*>0.05). Total GFAP protein in the whole co-culture system was quantified via Western blotting further confirmed the similar result (Figure 4D, *p*>0.05).

### Proliferation and differentiation of tumor cells in NSC-condition medium

**Figure 5:**
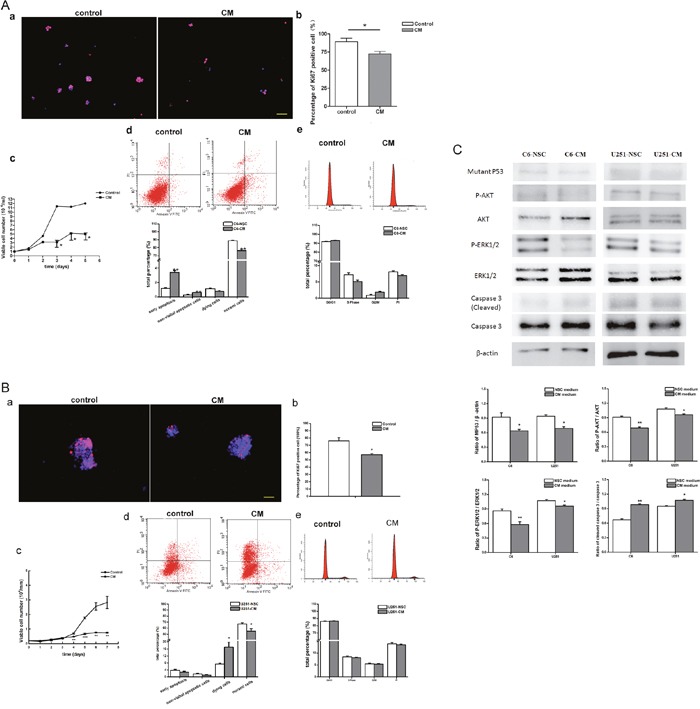
Survival and proliferation of tumor cell in the indirectly co-culture system **(A)** Survival and proliferation of C6 cells in NSC condition medium. **(B)** Survival and proliferation of U251 cells in NSC condition medium. a. Immunocytochemistry staining showed the ki67 positive proliferating cells. b. The percentage of ki67 positive cells. c. Growth curve of tumor cells. d. Apoptosis and necrosis of tumor cells, detected by FACS. e. Cell cycle distribution of tumor cells, analyzed by FACS. **(C)** Expression and phosphorylation of AKT and ERK1/2, the level of p53 and caspase 3, detected by Western blot. DAPI used for cell nuclei staining showed in blue, ki67 showed in red. CM means NSC condition medium. Scale bar=50 μm.*p<0.05, **p<0.01 and ***p<0.001.

Besides of the above directly co-culture system, an indirectly co-culture system was set up by culturing C6 and U251 cells in NSC-condition medium (NSC-CM) to further explore the effect of NSCs on tumor cell behaviors. After 3 days culturing in NSC-CM, cell proliferation as showed by the percentage of the Ki67 immune reactive cells was significantly inhibited. Approximately 72% of C6 cells were Ki67^+^ proliferating cells in NSC-CM, which is significantly lower than that in the control group (89%, Figure 5A). Similar result was found in U251 cells. The percentage of the Ki67^+^ proliferating cells significantly reduced from 76% to 57% (Figure 5B). Besides the declined growth of C6 and U251 cells, as showed by growth curve (Figure 5Ac and 5Bc), elevated early apoptosis of C6 (Figure 5Ad) and necrosis of U251 (Figure 5Bd) were also observed. However, no significant reduction was observed regarding cell cycle distribution (Figure 5Ae and 5Be). In coincidence with the above results, phosphorylation of AKT and ERK1/2 significantly declined, the level of mutant p53 decreased while the expression of caspase-3 increased dramatically (Figure 5C).

**Figure 6:**
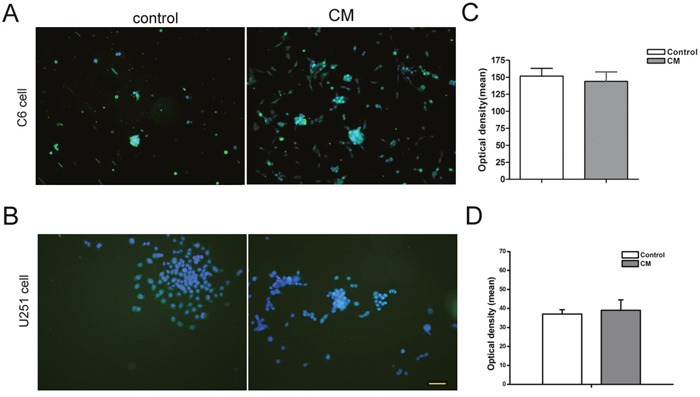
Differentiation of tumor cell in the indirectly co-culture system **(A** and **B)** Immunocytochemistry staining showed the GFAP positive cells. **(C** and **D)** The intensity of GFAP fluorescent assay showed that there was no significant difference between different groups. DAPI used for cell nuclei staining was showed in blue, GFAP showed in green. CM means condition medium. Scale bar=50 μm.

Regarding the maturational differentiation of C6 and U251 cells in this indirectly co-culture system, no significant difference was observed between different groups (Figure 6, *p*>0.05).

### Invasive ability and migration of tumor cells in co-cultured system

**Figure 7:**
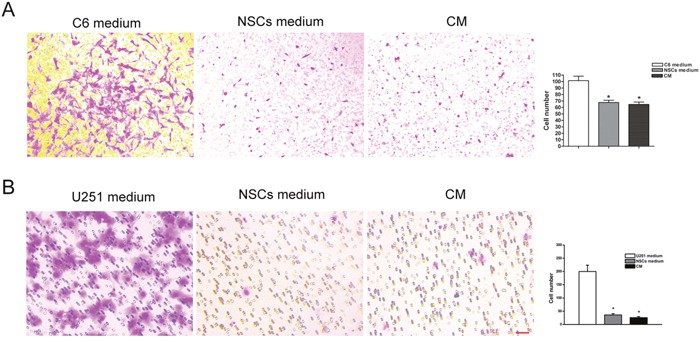
Invasive ability of tumor cell in the co-cultured system **(A)** Significantly reduced number of C6 cell passed through the transwell membrane, as compared with that cultured in C6 cell medium. **(B)** Significantly reduced number of U251 cell passed through the transwell membrane, as compared with that cultured in U251 cell medium. Cells underneath the transwell membrane were stained with crystal violet and showed in purple. C6 medium and U251 medium mean their original medium contained DMEM and 10% FBS, NSCs medium means NSC serum free medium. CM means condition medium. Scale bar = 50 μm. *p<0.05.

For the observation of cell invasion, C6 and U251 cells were co-cultured with NSCs both directly and in-directly (NSC-CM). Cell invasion was tested using transwell assay. The result showed that a significantly reduced number of cells, both C6 (Figure 7A) and U251 (Figure 7B), passed through the transwell membrane (*p*<0.05).

**Figure 8:**
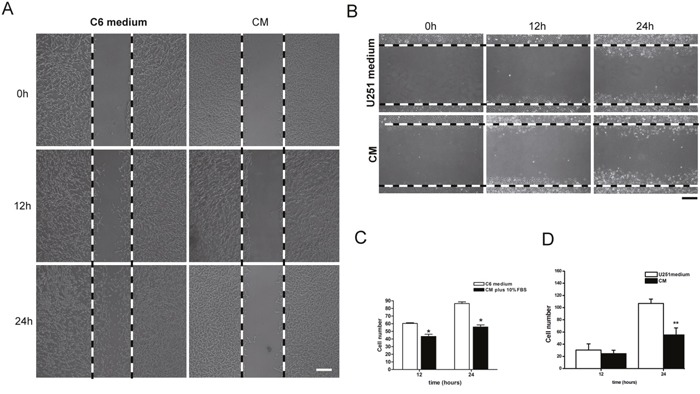
Migration of tumor cell in the co-cultured system **(A)** After 12 and 24 hrs culture, significantly less numbers of C6 cells migrated into the scraped area. **(B)** After 12 and 24 hrs culture, significantly less numbers of U251 cells migrated into the scraped area. **(C)** The number of C6 cells in the scraped space. **(D)** The number of U251 cells in the scraped space. CM means NSC condition medium. Scale bar =50 μm (A) and 25 μm (B). *p<0.05.

Cell migration was detected by wound healing test. Tumor cells, both C6 and U251 cells, were cultured in NSC-CM for 12 hrs and 24 hrs. Results showed that a significantly reduced numbers of C6 and U251 cells migrated into the scraped space (Figure 8, *p*<0.05).

### Growth of tumor cells in nude mouse

**Figure 9:**
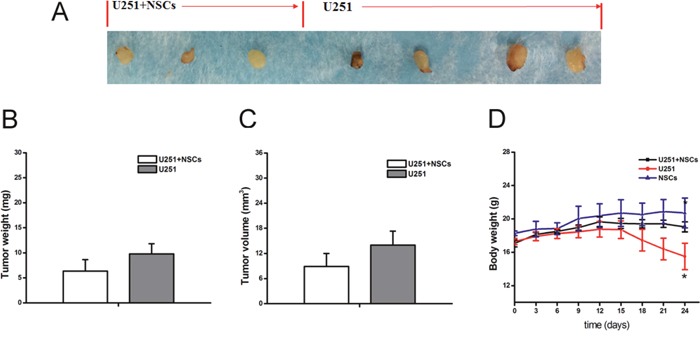
Tumor growth in nude mice **(A)** Different sizes of tumors were observed in all mice after tumor cell injection. **(B** and **C)** The overall weight and volume of tumors from the mice injected with U251 cell alone slightly higher than that with U251+NSCs injection. **(D)** Body weight of tumor bearing mice showed the significant reduction after U251 cells injection. *p<0.05.

The *in vivo* inhibition of NSCs on tumor growth was further investigated. U251 cells were co-cultured with NSCs and subcutaneously injected to nude mice. Tumors formed slowly after injection of U251 cells alone (4/4) and U251 cells with NSCs (3/4). No significant reduction of the overall weight and volume of tumors were observed. However, the body weight of nude mice injected with U251 cells significantly declined since the 15^th^ day after injection, and it was significantly less than that injected with U251 cells and NSCs (Figure 9). In addition, all nude mice injected with U251 cells dead at 24^th^ day after tumor cells xenografting while others were survived.

## DISCUSSION

Here in order to mimic the interaction between tumor cells and NSCs *in situ*, we co-cultured rat and human glioma cells with NSCs directly and indirectly *in vitro*. Our results showed that the survival, growth, proliferation and migration/invasion of tumor cell significantly declined in the co-culture system. Consistent with this, the level of mutant p53, phosphorylation of AKT and ERK1/2 reduced while caspase-3 was up-regulated significantly. No dramatic change was observed regarding glioma cell maturational differentiation. Moreover, *in vivo* observation showed that the survival status and time of tumor-bearing mice was promoted when glioma cells were subcutaneously injected with NSCs.

NSCs displayed extensive tropism for pathology in adult brain [19]. After implanted either intracranial or intravascularly, NSCs migrated through normal tissue targeting the tumor cells and distributed extensively throughout the tumor bed. This indicated that NSCs might directly interact with tumor cells *in situ*. In the current study, rat glioma cells C6 and human glioma cells U251 were directly co-cultured with rat embryonic NSCs to allow a close interaction between NSCs and tumor cells [28]. In comparison with tumor cells cultured in their original medium and serum free NSCs medium, after co-cultured with NSCs, tumor cell viability, proliferation, invasion and migration were significantly attenuated. The reduction seems to be independent on the amount of NSCs. In order to overcome the main disadvantage of direct co-culture system, that is the difficulty in evaluating the effect that the one group of cells has on the other group of cells, an indirect co-culture system by culturing tumor cells in NSCs condition medium was also used. Almost identical results have been obtained. In addition, the possible effects of normal NSCs on tumor genesis and progress were also investigated after subcutaneously injected U251 cells and NSCs into nude mice. Although the tumor formation was not affected, the survival status of tumor bearing mice was significantly promoted.

It has been previously demonstrated that astrocytoma associated neural precursors could induce tumor cell death via releasing endovanilloids [29]. Here our results further demonstrated that normal NSCs could also reduce the glioma genesis and growth somehow. These results were confirmed by the alteration of MAPK and PI3K signals and the enhanced apoptosis and necrosis of tumor cells.

Forcing glioma cells to terminal differentiation has also been considered as an important therapeutic approach [30]. In the current study, the maturational differentiation of C6 and U251 cells after co-cultured with normal NSCs was detected. Neither the percentage of GFAP positive cells nor the protein level of GFAP in the co-culture system was significantly changed. It suggested that in the current system, normal NSCs did not offer an influence on glioma cells maturation.

One thing might be worth mentioning is that glioma cells were cultured in serum free NSC medium prior to the co-culture with NSCs. We noticed that tumor cells grew into 3 dimensional spheres and almost all of the cells were nestin positive (Supplementary Figure 1). This indicated that those cells probably were glioma stem cells (GSCs), which are subpopulation of cells in malignant glioma that display stem cell properties [31–34]. This may somehow explain the low invasion of C6 and U251 cells in NSCs medium. Most importantly, it also indicated that the anti-tumor progress effect of NSCs in the co-culture system may through directly affecting the GSCs, rather than glioma cells. Further study to clarify the interaction between normal NSCs and GSCs is urgently needed.

Taking all the results together, we concluded that normal NSCs possessed an efficient anti-glioma property via inhibiting tumor cell viability, proliferation, invasion and migration. Consequently, NSC could be a very high potential candidate for glioma treatment and GSCs might be the direct target.

## MATERIALS AND METHODS

### Culture and *in vitro* labeling of tumor cells

Rat glioma cell line- C6 and human glioma cell line-U251 were purchased from ATCC (American Type Culture Collection, USA). Cells were cultured in the tumor cell medium which contained DMEM (Dulbecco's modified Eagle medium, Invitrogen, USA) and 10% FBS (Gibco, USA) after defrost. In order to set up the co-culture system, upon sub-culturing, C6 and U251 cells were maintained in DMEM medium with 1% FBS for 6 days, and then were sub-cultured in NSCs growth medium to let them adapt to the serum free condition before co-culture.

Fluorescent carbocyanine dye CM-DiI (Molecular Probes) was used to label C6 and U251 cells. After washing with PBS, cells were incubated with CM-DiI at a concentration of 5 μmol/L for 5 min at 37°C and then for 15 min at 4°C in dark. The labeled cells were visualized under the fluorescent microscope and cell counting was performed to evaluate the labeling efficiency.

### Isolation and culture of NSCs

Pregnant female Sprague-Dawley rats were provided by Experimental Animal Center, Xi’an Jiaotong University Health Science Center. All procedures involving animal work conformed to the ethical guidelines of the NIH Regulations for Experimentation on Laboratory Animals and set out by the Xi’an Jiaotong University.

NSCs were isolated from cerebral cortex of SD rat embryos on E14.5 days following the protocols of Gage FH and optimized in our laboratory [35, 36]. Cells were cultured in serum free growth medium which contained DMEM/F12, 10 ng/ml bFGF, 20 ng/ml EGF, 1% penicillin, 1% streptomycin, 1% N2, and 2% B27 supplement (all from Invitrogen, Carlsbad, US) and 2.5 μg/ml heparin (Sigma, St. Louis, US). NSCs differentiation medium contained DMEM/F12 (1:1), 1% N2, 2% B27 supplement, 100 U/mL penicillin, 100 μg/mL streptomycin and 1% FBS. Primary cultured cells were sub-cultured every 5 days. NSCs identification was performed via immunocytochemistry staining. Primary antibodies, including monoclonal mouse anti-nestin (1:200, Millipore, Temecula, US), monoclonal mouse anti-β tubulin III (1:200, Millipore, Temecula, US) and polyclonal rabbit anti-GFAP (1:500, Santa Cruz, CA, USA) were used.

### Establishment of the tumor cell and NSCs co-culture system

Both directly co-culture system and indirectly co-culture system were set up in the current study to mimic the *in vivo* glioma microenvironment. For the directly co-culture system, C6 and U251 cells were mixed with different numbers of primary cultured NSCs on passage number 2 or 3 and then cultured in serum free NSCs growth medium. For the indirectly co-culture system, C6 and U251 cells were cultured in NSC-CM. NSC-CM consisted of 50% of fresh NSC growth medium and 50% of the medium collected from NSCs culture system on the 5th day.

### Cell viability and proliferation assay

CCK-8 assay, growth curve plotting, flow cytometer analysis (FACS) and Ki67 immunostaining were used to investigate tumor cell viability and proliferation. For CCK-8 assay, approximately 2,000 of tumor cells were mixed with different numbers of NSCs (1,000, 2,000, 8,000 and 16,000, respectively) and seeded in 96-well plates (Costar). According to the manufactory illustration, before testing, about 10 μl CCK-8 solution (water-soluble tetrazolium salt WST-8 [2-(2-methoxy-4-nitrophenyl)-3-(4-nitrophenyl)-5-(2,4-disulfophenyl)-2H-tetrazolium]) was added to each of three individual wells on 1, 3, 5 and 7 days *in vitro* (DIV), respectively. WST-8 will be reduced by dehydrogenases in cells to give an orange colored product (formazan). One hour later, the amount of the formazan dye, which is directly proportional to the number of living cells, was measured with Universal Microplate Spectrophotometer (QuantTM BioTek) at 450 nm. In addition, equivalent numbers of tumor cells and NSCs were cultured separately in the same condition and the OD450 of tumor cells plus OD450 of NSCs was considered as control. Three independent experiments were performed. The inhibitory rate of cell viability was calculated as follow.

Inhibitory rate (%) = (OD450 of C6 cells + OD450 of NSCs - OD450 of co-cultured cells)/OD450 of C6 cells × 100 %.

For growth curve plotting, 5,000 of C6 or U251 cells were cultured with NSCs in (1:1) in serum free NSC growth medium. The number of all cells as well as the CM-DiI labeled tumor cells was counted every day for 7 days. Ki67 immunostaining was applied to identify the cell proliferation (polyclonal mouse anti-ki67, 1:300, Millipore, Temecula, US). In addition, cell cycle distribution and apoptosis of tumor cells were examined by FACS. Approximately 1×10^6^ of C6 or U251 cells were cultured in NSC-CM for 3 days and annexin V-FITC/PI apoptotic detection kit (BD, US) was used.

### Assessment of tumor cell differentiation

Differentiation of C6 and U251 cells was investigated by both immunocytochemistry staining and Western blot assay. For immunostaining, C6 and U251 cells were either co-cultured with different numbers of NSCs in differentiation medium or in NSC-CM for 7 days and then fixed with ice-cold 4% paraformaldehyde (PFA) at room temperature for 30 min. Immunocytochemistry staining was performed following the standard protocol and optimized in author's laboratory [36]. Primary antibodies were diluted in PBS containing 2% normal goat serum (NGS). Blocking solution contained 5% NGS and 0.25% Triton X-100 in PBS. FITC-conjugated or TRITC-conjugated goat anti-mouse/anti-rabbit IgG (1: 400; CWBIO, Peking, CN) was used as secondary antibody. Cell nuclei were counterstained with DAPI-containing mounting media (Sigma, St. Louis, Mo, USA). Cells were visualized under a fluorescent microscope (Olympus BX57) equipped with a DP70 digital camera and the DPManager (DPController, Olympus) software. For the negative control, primary antibody was replaced by blocking buffer. The intensity of fluorescent indicating the percentage of GFAP positive cells was measured by image-pro plus 5.0 software.

Differentiation of C6 and U251 cells was also quantified with Western blot assay. All co-cultured cells (C6 and NSCs or U251 and NSCs) were collected, then rinsed with ice-cold PBS and re-suspended with cold RIPA Lysis Buffer. After 5 min on ice, lysates were harvested and centrifuged at 12,000g for 10 min. Total protein concentration of lysates was measured using NanoDrop ND-1000. Soluble protein was separated on 10% polyacrylamide gels and blotted onto nitrocellulose membrane by standard procedures. Then the membranes were incubated with polyclonal antibody GFAP (1:5,000, Abcam) and β-actin (1:20,000, Boster, China) at 4°C overnight, washed with Tris Buffered Saline with Tween (TBST) and incubated with the alkaline phosphatase (AP)-labeled secondary antibody for 2 hrs at room temperature. The membranes were visualized by an enhanced chemiluminescence (ECL) method and the data were analyzed with Image J (version 1.61).

### Observation of tumor cell invasion and migration

Transwell assay was used to investigate cell invasion. In the directly co-culture system, approximately 0.5×10^5^ of tumor cells with 2×10^5^ of NSCs were suspended in 500 μl NSC growth medium and co-cultured in the upper compartment of transwell (with 8 μm pore membranes, Corning Incorporated, Corning, NY, USA). In the indirect co-culture system, 0.5×10^5^ of tumor cells were cultured in NSC-CM, NSC growth medium and tumor cell medium (DMEM+10% FBS) in the upper compartment. Another 500 μl NSCs growth medium was added in the lower compartment. After 24 hrs incubation, the insert was removed and the cells in upper part were cleaned with a cotton swab. Then the cells underneath the membrane were fixed with 4% PFA for 20 min at room temperature and stained with 0.1% crystal violet for 10 min. Cell number in 3 individual fields of each insert was counted. The result from equivalent numbers of tumor cells and NSCs cultured separately in NSCs growth medium was taken as control.

Cell migration was tested by wound healing test. C6 and U251 cells were seeded onto poly-L-lysine coated coverslips in 6-well plate and cultured in the tumor cell medium and NSC-CM plus 10% FBS, which is used to facilitate cell attachment, respectively. When cells grew up to 80% ~ 90% confluence, a scraped wound was made by the fine end of pipette tip. After another 12 hrs and 24 hrs culture, the number of cells migrated into the scraped area was counted.

### Assessment of the possible mechanisms of tumor cells biological behavior changes

In order to further investigate the possible mechanisms of NSCs’ effect on tumor cell behavior changes, expression of mutant p53, caspase-3 and the phosphorylation status of ERK1/2, AKT were detected by Western blot assay. Rabbit monoclonal anti-mutant p53 (1:1,000, abcam, USA), rabbit monoclonal anti-GFAP (1:2,000, Millopore, USA), rabbit polyclonal anti-caspase 3 (1:1,000, Cell Signaling, USA), rabbit polyclonal anti-AKT (1:1,000, Cell Signaling, USA), rabbit monoclonal anti-p-AKT (1:1,000, Cell Signaling, USA), rabbit monoclonal anti-ERK1/2 (1: 1,000, Cell Signaling USA), rabbit monoclonal anti-p-ERK 1/2 (1: 1,000, Cell Signaling USA) were used.

### Subcutaneously implantation of tumor cells in nude mice

In order to further assess the inhibitory effect of NSCs on tumor genesis, nude mice (12 in total) were used and were divided into 3 groups. About 4×10^6^ of U251 cells were subcutaneously injected into the left armpits (n=4, named group I and labeled as U251). Same amount of U251 cells were co-cultured with NSCs (1:1) and then subcutaneously injected into the right armpits (n=4, named group II and labeled as U251+NSCs). Mice injected with NSCs (4×10^6^) were taken as control (n=4). Then tumor growth was observed every 3 days until 24 days after injection. The body weight of nude mice was also monitored. Before sacrifice, the xenograft were stripped out and weighed, the volumes were measured using the formula: V (mm^3^) = (a) × (b^2^/2), where ‘a’ is longest tumor diameter and ‘b’ is the shortest tumor diameter.

### Statistics analysis

All experiments were performed in triplicate. The data was shown as mean ± SE, and analyzed with the SPSS 18.0 software. Student's T test and one-way ANOVA were used for multiple comparison and a *p* value <0.05 was considered to be statistically significant.

## SUPPLEMENTARY MATERIALS FIGURE



## References

[1] Stupp R, Mason WP, van den Bent MJ, Weller M, Fisher B, Taphoorn MJ, Belanger K, Brandes AA, Marosi C, Bogdahn U, Curschmann J, Janzer RC, Ludwin SK (2005). Radiotherapy plus concomitant and adjuvant temozolomide for glioblastoma. N Engl J Med.

[2] Goodenberger ML, Jenkins RB (2012). Genetics of adult glioma. Cancer Genet.

[3] Nabors LB, Portnow J, Ammirati M, Brem H, Brown P, Butowski N, Chamberlain MC, DeAngelis LM, Fenstermaker RA, Friedman A, Gilbert MR, Hattangadi-Gluth J, Hesser D (2014). Central nervous system cancers, version 2.2014. Featured updates to the NCCN Guidelines. J Natl Compr Cancer Netw.

[4] Kaluzova M, Bouras A, Machaidze R, Hadjipanayis CG (2015). Targeted therapy of glioblastoma stem-like cells and tumor non-stem cells using cetuximab-conjugated iron-oxide nanoparticles. Oncotarget.

[5] Karpel-Massler G, Bâ M, Shu C, Halatsch ME, Westhoff MA, Bruce JN, Canoll P, Siegelin MD (2015). TIC10/ONC201 synergizes with Bcl-2/Bcl-xL inhibition in glioblastoma by suppression of Mcl-1 and its binding partners *in vitro* and *in vivo*. Oncotarget.

[6] Zhou W, Cheng L, Shi Y, Ke SQ, Huang Z, Fang X, Chu CW, Xie Q, Bian XW, Rich JN, Bao S (2015). Arsenic trioxide disrupts glioma stem cells via promoting PML degradation to inhibit tumor growth. Oncotarget.

[7] Wang J, Yan Z, Liu X, Che S, Wang C, Yao W (2016). Alpinetin targets glioma stem cells by suppressing Notch pathway. Tumour Biol.

[8] Aboody KS, Najbauer J, Danks MK (2008). Stem and progenitor cell-mediated tumor selective gene therapy. Gene Ther.

[9] English K, Wood KJ (2011). Immunogenicity of embryonic stem cell-derived progenitors after transplantation. Curr Opin Organ Transplant.

[10] Jones BJ, McTaggart SJ (2008). Immunosuppression by mesenchymal stromal cells: from culture to clinic. Exp Hematol.

[11] Einstein O, Ben-Hur T (2008). The changing face of neural stem cell therapy in neurologic diseases. Arch Neurol.

[12] Li S, Tokuyama T, Yamamoto J, Koide M, Yokota N, Namba H (2005). Bystander effect-mediated gene therapy of gliomas using genetically engineered neural stem cells. Cancer Gene Ther.

[13] Davis AA, Temple S (1994). A self-renewing multipotential stem cell in embryonic rat cerebral cortex. Nature.

[14] Gage FH (2000). Mammalian neural stem cells. Science.

[15] Chen L, Qiu R, Li L, He D, Lv H, Wu X, Gu N (2014). The role of exogenous neural stem cells transplantation in cerebral ischemic stroke. J Biomed Nanotechnol.

[16] Assinck P, Duncan GJ, Hilton BJ, Plemel JR, Tetzlaff W (2017). Cell transplantation therapy for spinal cord injury. Nat Neurosci.

[17] Marsh SE, Blurton-Jones M (2017). Neural stem cell therapy for neurodegenerative disorders: the role of neurotrophic support. Neurochem Int.

[18] Aboody KS, Najbauer J, Metz MZ, D'Apuzzo M, Gutova M, Annala AJ, Synold TW, Couture LA, Blanchard S, Moats RA, Garcia E, Aramburo S, Valenzuela VV (2013). Neural stem cell-mediated enzyme/prodrug therapy for glioma: preclinical studies. Sci Transl Med.

[19] Aboody KS, Brown A, Rainov NG, Bower KA, Liu S, Yang W, Small JE, Herrlinger U, Ourednik V, Black PM, Breakefield XO, Snyder EY (2000). Neural stem cells display extensive tropism for pathology in adult brain: evidence from intracranial gliomas. Proc Natl Acad Sci U S A.

[20] Yip S, Sabetrasekh R, Sidman RL, Snyder EY (2006). Neural stem cells as novel cancer therapeutic vehicles. Eur J Cancer.

[21] Ahmed AU, Alexiades NG, Lesniak MS (2010). The use of neural stem cells in cancer gene therapy: predicting the path to the clinic. Curr Opin Mol Ther.

[22] Barresi V, Belluardo N, Sipione S, Mudo G, Cattaneo E, Condorelli DF (2003). Transplantation of prodrug-converting neural progenitor cells for brain tumor therapy. Cancer Gene Ther.

[23] Namba H, Kawaji H, Yamasaki T (2016). Use of genetically engineered stem cells for glioma therapy. Oncol Lett.

[24] Achanta P, Sedora Roman NI, Quiñones-Hinojosa A (2010). Gliomagenesis and the use of neural stem cells in brain tumor treatment. Anticancer Agents Med Chem.

[25] Li S, Tokuyama T, Yamamoto J, Koide M, Yokota N, Namba H (2005). Potent bystander effect in suicide gene therapy using neural stem cells transduced with herpes simplex virus thymidine kinase gene. Oncology.

[26] Liu S, Yin F, Zhao M, Zhou C, Ren J, Huang Q, Zhao Z, Mitra R, Fan W, Fan M (2016). The homing and inhibiting effects of hNSCs-BMP4 on human glioma stem cells. Oncotarget.

[27] Glass R, Synowitz M, Kronenberg G, Walzlein JH, Markovic DS, Wang LP, Gast D, Kiwit J, Kempermann G, Kettenmann H (2005). Glioblastoma-induced attraction of endogenous neural precursor cells is associated with improved survival. J Neurosci.

[28] Pandurangan M, Hwang I (2014). Application of cell co-culture system to study fat and muscle cells. Appl Microbiol Biotechnol.

[29] Stock K, Kumar J, Synowitz M, Petrosino S, Imperatore R, Smith ES, Wend P, Purfürst B, Nuber UA, Gurok U, Matyash V, Wälzlein JH, Chirasani SR (2012). Neural precursor cells induce cell death of high-grade astrocytomas through stimulation of TRPV1. Nat Med.

[30] Guichet PO, Bieche I, Teigell M, Serguera C, Rothhut B, Rigau V, Scamps F, Ripoll C, Vacher S, Taviaux S, Chevassus H, Duffau H, Mallet J (2013). Cell death and neuronal differentiation of glioblastoma stem-like cells induced by neurogenic transcription factors. Glia.

[31] Benda P, Lightbody J, Sato G, Levine L, Sweet W (1968). Differentiated rat glial cell strain in tissue culture. Science.

[32] Chao CC, Kan D, Lo TH, Lu KS, Chien CL (2015). Induction of neural differentiation in rat C6 glioma cells with taxol. Brain Behav.

[33] Zhou XD, Wang XY, Qu FJ, Zhong YH, Lu XD, Zhao P, Wang DH, Huang QB, Zhang L, Li XG (2009). Detection of cancer stem cells from the C6 glioma cell line. J Int Med Res.

[34] Zheng X, Shen G, Yang X, Liu W (2007). Most C6 cells are cancer stem cells: evidence from clonal and population analyses. Cancer Res.

[35] Gage FH, Coates PW, Palmer TD, Kuhn HG, Fisher LJ, Suhonen JO, Peterson DA, Suhr ST, Ray J (1995). Survival and differentiation of adult neuronal progenitor cells transplanted to the adult brain. Proc Natl Acad Sci U S A.

[36] Lu H, Jiao Q, Wang Y, Yang Z, Feng M, Wang L, Chen X, Jin W, Liu Y (2013). The mental retardation-associated protein srGAP3 regulates survival, proliferation, and differentiation of rat embryonic neural stem/progenitor cells. Stem Cells Dev.

