# Supplementary material for: The inhibiting effect of neural stem cells on proliferation and invasion of glioma cells
Source: Oncotarget. 2017 Aug 14;8(44):76949–60. doi: 10.18632/oncotarget.20270 (PMC5652754; doi:10.18632/oncotarget.20270)
Supplement: Supplementary file 1 [file oncotarget-08-76949-s001.pdf]

The inhibiting effect of neural stem cells on proliferation and invasion of glioma cells

SUPPLEMENTARY MATERIALS

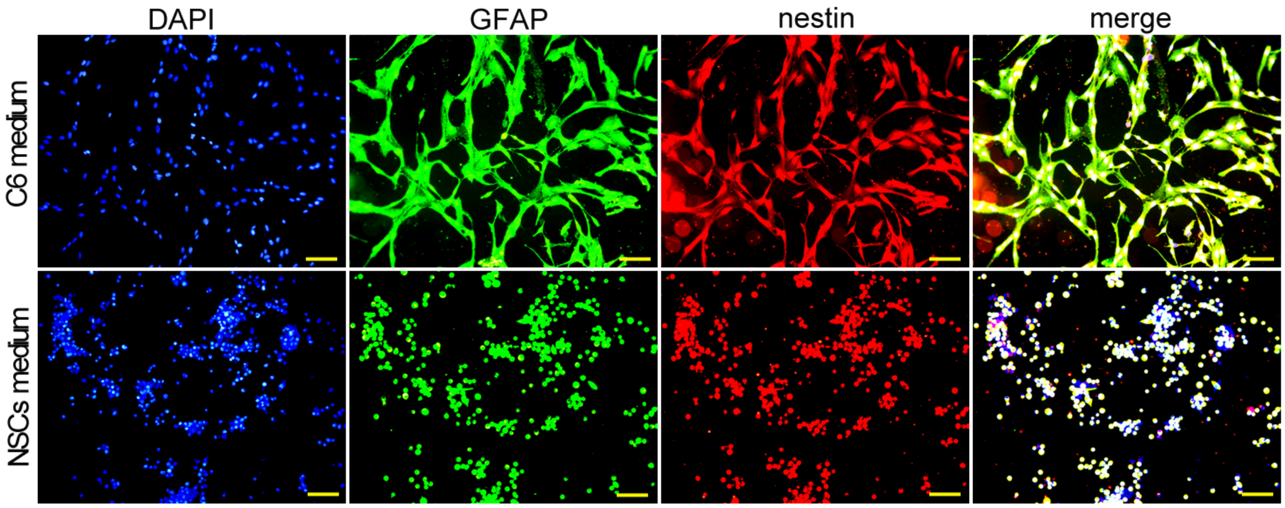

Supplementary Figure 1: Almost all of C6 glioma cells were nestin positive both in their original medium and serum free NSC medium.
